# Unraveling immunological heterogeneity in recalcitrant AIH-PBC/PSC overlap syndromes: from molecular crosstalk to precision therapeutics

**DOI:** 10.3389/fimmu.2026.1819131

**Published:** 2026-04-30

**Authors:** Zefeng Zhang

**Affiliations:** Wuxi Medical College, Jiangnan University, Wuxi, China

**Keywords:** autoimmune hepatitis, immunological heterogeneity, overlap syndrome, precision medicine, primary biliary cholangitis

## Abstract

The clinical management of autoimmune liver diseases is frequently complicated by the concomitant presentation of hepatitic and cholestatic features, an entity clinically designated as Overlap Syndrome. While the majority of patients respond to conventional combinatorial therapy involving corticosteroids and ursodeoxycholic acid, a significant subset exhibits a recalcitrant phenotype characterized by biochemical non-response and rapid fibrotic progression. The pathogenesis driving this therapeutic resistance remains incompletely defined, though emerging evidence implicates a distinct immunological architecture rather than a simple superposition of two diseases. This review delineates the multidimensional immunopathogenesis of AIH-PBC and AIH-PSC overlap syndromes with a specific focus on the interface between adaptive immunity and biliary epithelial senescence. We critically examine recent single-cell transcriptomic data that reveal specific pro-inflammatory macrophage niches and exhausted T-cell signatures unique to the overlap phenotype. Furthermore, we discuss the molecular crosstalk involving bile acid signaling and inflammatory cascades that perpetuates liver injury despite standard immunosuppression. Finally, we propose a paradigm shift towards precision medicine by evaluating the rationale for emerging biologic agents, including JAK inhibitors and B-cell depleting therapies, in the management of difficult-to-treat overlap syndromes.

## Graphical Abstract

**Figure:**
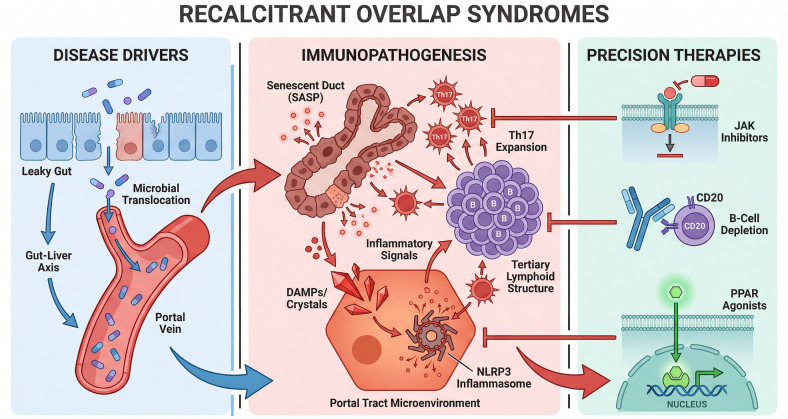

## 1 Introduction

### 1.1 The clinical spectrum and therapeutic impasse

Autoimmune liver diseases encompass a broad spectrum of hepatobiliary pathologies predominantly classified into autoimmune hepatitis (AIH), primary biliary cholangitis (PBC), and primary sclerosing cholangitis (PSC). Although these entities are traditionally distinguished by specific serological markers and histological patterns, clinical reality often defies rigid categorization (6). Overlap syndromes represent a distinct diagnostic cluster where patients manifest simultaneous or sequential features of both hepatocelluar and cholestatic injury (7). Among these, the AIH-PBC variant constitutes the most prevalent form, whereas AIH-PSC overlap presents unique challenges particularly in the pediatric population and young adults (8).

Importantly, overlap syndromes do not represent a monolithic entity but exhibit profound fundamental heterogeneity. AIH-PBC and AIH-PSC overlaps demonstrate markedly divergent immunopathogenic mechanisms and genetic susceptibility loci. Classical AIH-PBC overlap predominantly affects middle-aged females, characterized by AMA positivity and a hybrid T-cell/B-cell destructive signature in small bile ducts. In contrast, AIH-PSC overlap—often diagnosed as Autoimmune Sclerosing Cholangitis (ASC) in pediatric populations—presents with large duct involvement, pANCA positivity, and a dominant pathogenetic link to inflammatory bowel disease (IBD). Furthermore, clinical trajectories vary significantly depending on whether the overlap features present as a *de novo* simultaneous condition or as sequential phenotypic evolution, such as treatment-induced transformation where a well-controlled AIH gradually acquires cholestatic features over years.

The prevailing therapeutic strategy relies on the empiric combination of ursodeoxycholic acid and corticosteroids or azathioprine. This regimen successfully induces biochemical remission in the majority of cases (9). However, clinical data indicate that approximately 10% to 20% of patients fail to achieve normalization of liver enzymes or histological resolution (10). This recalcitrant cohort faces a significantly elevated risk of progression to end-stage liver disease and requirement for liver transplantation compared to patients with pure AIH or PBC (1, 11). The persistence of inflammation despite broad-spectrum immunosuppression suggests that the immunopathogenic drivers in these patients may differ fundamentally from those in responsive individuals.

### 1.2 Divergent immunological mechanisms

Current understanding of overlap syndromes has evolved from viewing them as a coincidence of two diseases to recognizing them as a continuum of immune dysregulation (12). The distinct genetic susceptibility loci identified in genome-wide association studies, such as HLA-DRB1 and IL12A, point toward a shared predisposition but divergent effector pathways (13). In the refractory overlap phenotype, the immune microenvironment is hypothesized to undergo substantial remodeling. Recent advances in single-cell RNA sequencing have begun to unravel this heterogeneity by identifying specific subsets of cytotoxic CD8^+^ T cells and clonally expanded B cells that reside within the fibrotic niche (3, 14). Furthermore, the interaction between injured cholangiocytes and the hepatic immune system creates a self-perpetuating inflammatory loop. Cholangiocytes in these patients are not merely passive targets but active drivers of inflammation through the secretion of senescence-associated secretory phenotype factors and chemokines including CCL2 and CX3CL1 (15).

### 1.3 The imperative for mechanism-based therapeutics

The limitation of current steroid-based protocols necessitates the exploration of targeted biologic therapies. The identification of key molecular nodes, such as the JAK-STAT signaling pathway and B-cell survival factors, offers new avenues for intervention (16). Several novel agents originally developed for other autoimmune conditions are now being repurposed for refractory autoimmune liver diseases with promising preliminary results (5, 17).

### 1.4 Scope of review

In this review, we dissect the complex immunological landscape of AIH-PBC and AIH-PSC overlap syndromes. We specifically focus on the cellular and molecular mechanisms that underpin therapeutic resistance. We first categorize the immunological heterogeneity revealed by high-dimensional omics technologies. Subsequently, we examine the molecular crosstalk between the gut-liver axis and local immune responses (4). Finally, we provide a mechanism-based rationale for the deployment of second-line therapies, aiming to bridge the gap between basic immunological insights and clinical precision management (2).

## 2 Decoding the immune microenvironment: cellular drivers of recalcitrance

The establishment of a chronic inflammatory niche in overlap syndromes relies on a complex interplay between innate and adaptive immune compartments. Unlike the singular autoimmune dominance observed in classic AIH or PBC, the overlap phenotype exhibits a hybrid immunological signature that may facilitate escape from standard immunosuppressive mechanisms (18).

### 2.1 T-helper plasticity and the Th17 axis

The balance between regulatory T cells (Tregs) and effector T cells constitutes the primary checkpoint for hepatic immune tolerance. In patients with recalcitrant overlap syndromes, the frequency and function of Tregs are often compromised, leading to an unchecked expansion of autoreactive effector clones (19). Of particular relevance is the phenomenon of T-helper cell plasticity, where Th17 cells undergo a lineage shift toward a Th1-like phenotype in the inflammatory milieu (20). These ex-Th17 cells retain their capacity to produce IL-17 while simultaneously secreting IFN-gamma, creating a dual-cytokine environment that is notably resistant to corticosteroid therapy (21). The enrichment of IL-17-producing cells in the portal tracts of AIH-PBC overlap patients correlates with the degree of bile duct destruction and interface hepatitis severity (22). Furthermore, the recruitment of these pathogenic T cells is mediated by the upregulation of CCL20 by injured cholangiocytes, establishing a feed-forward loop of inflammation that persists even when peripheral inflammation markers subside (23).

### 2.2 The B-cell compartment and tertiary lymphoid structures

**Figure 1:**
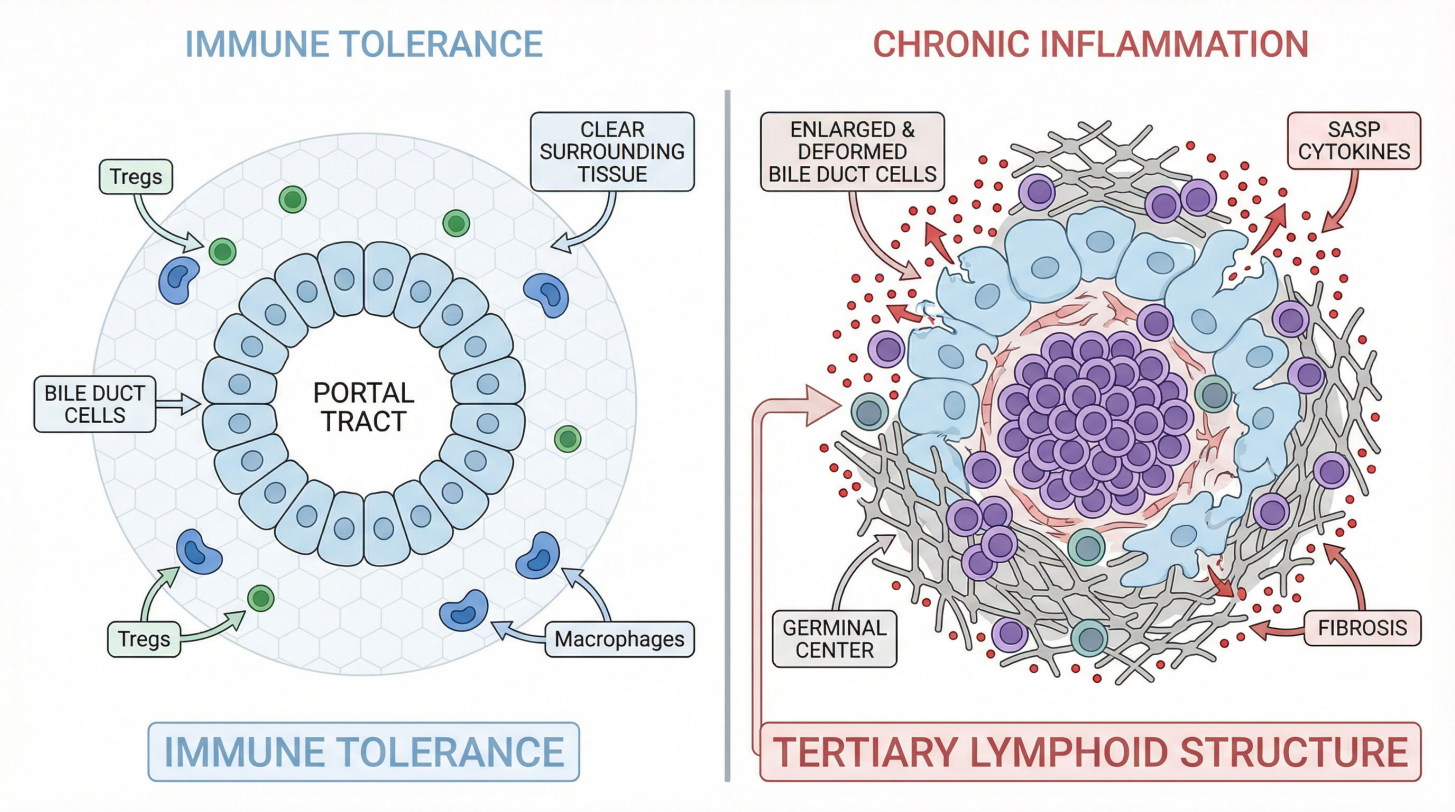
The immunological landscape of the hepatic microenvironment in responsive versus recalcitrant overlap syndromes. Left panel (Immune Tolerance): In the responsive or quiescent state, immune tolerance is successfully maintained within the hepatic niche. Intact cholangiocytes line the bile ducts, and resident macrophages (Kupffer cells) remain in a resting state. Regulatory T cells (Tregs) dominate the local microenvironment, effectively suppressing autoreactive responses and preserving tissue architecture without fibrotic remodeling. Right panel (Chronic Inflammation): Conversely, the recalcitrant (refractory) state is characterized by chronic inflammation and structural distortion. Senescent cholangiocytes acquire a senescence-associated secretory phenotype (SASP), releasing pro-inflammatory cytokines and chemokines. This milieu drives the local expansion of pathogenic Th17 cells and the formation of ectopic tertiary lymphoid structures (B-cell follicles), which perpetuate an auto-amplifying inflammatory loop. Concurrently, active fibrogenesis is orchestrated by myofibroblasts, resulting in dense periductal fibrosis and disruption of the limiting plate (interface hepatitis). Treg, regulatory T cell; SASP, senescence-associated secretory phenotype; Th17, T helper 17 cell.

While B cells are traditionally viewed through the lens of autoantibody production, their role in overlap syndromes extends significantly beyond humoral immunity. B cells function as potent antigen-presenting cells that modulate T-cell responses within the hepatic microenvironment (24). In distinct subsets of AIH-PBC overlap patients, histological analysis reveals the formation of tertiary lymphoid structures (TLS) within the portal triads (**Figure 1**) (25). While available evidence strongly supports TLS primarily as a robust biomarker of disease severity and advanced fibrosis, these ectopic germinal centers also provide a localized niche that facilitates local affinity maturation and clonal expansion of B cells, thereby sustaining the autoimmune response independently of systemic circulation (26). The presence of such organized lymphoid aggregates often signifies a refractory clinical course and poor response to standard first-line monotherapies (e.g., ursodeoxycholic acid for PBC or corticosteroids for AIH). Moreover, intrahepatic B cells in these patients express high levels of activation markers such as CD80 and CD86, suggesting their critical role in providing costimulatory signals to cytotoxic T lymphocytes (27). This non-humoral function of B cells suggests a potential mechanistic rationale for the efficacy of B-cell depletion therapies in cases where autoantibody titers do not correlate with disease activity, though further prospective clinical validation remains required (28).

## 3 Molecular crosstalk at the tissue interface

The progression from inflammation to fibrosis in overlap syndromes is driven by molecular crosstalk between infiltrating immune cells and resident parenchymal cells. This interaction occurs primarily at two distinct interfaces: the hepatocyte-lymphocyte interface and the cholangiocyte-lymphocyte interface.

### 3.1 The reactive cholangiocyte and senescence

**Figure 2:**
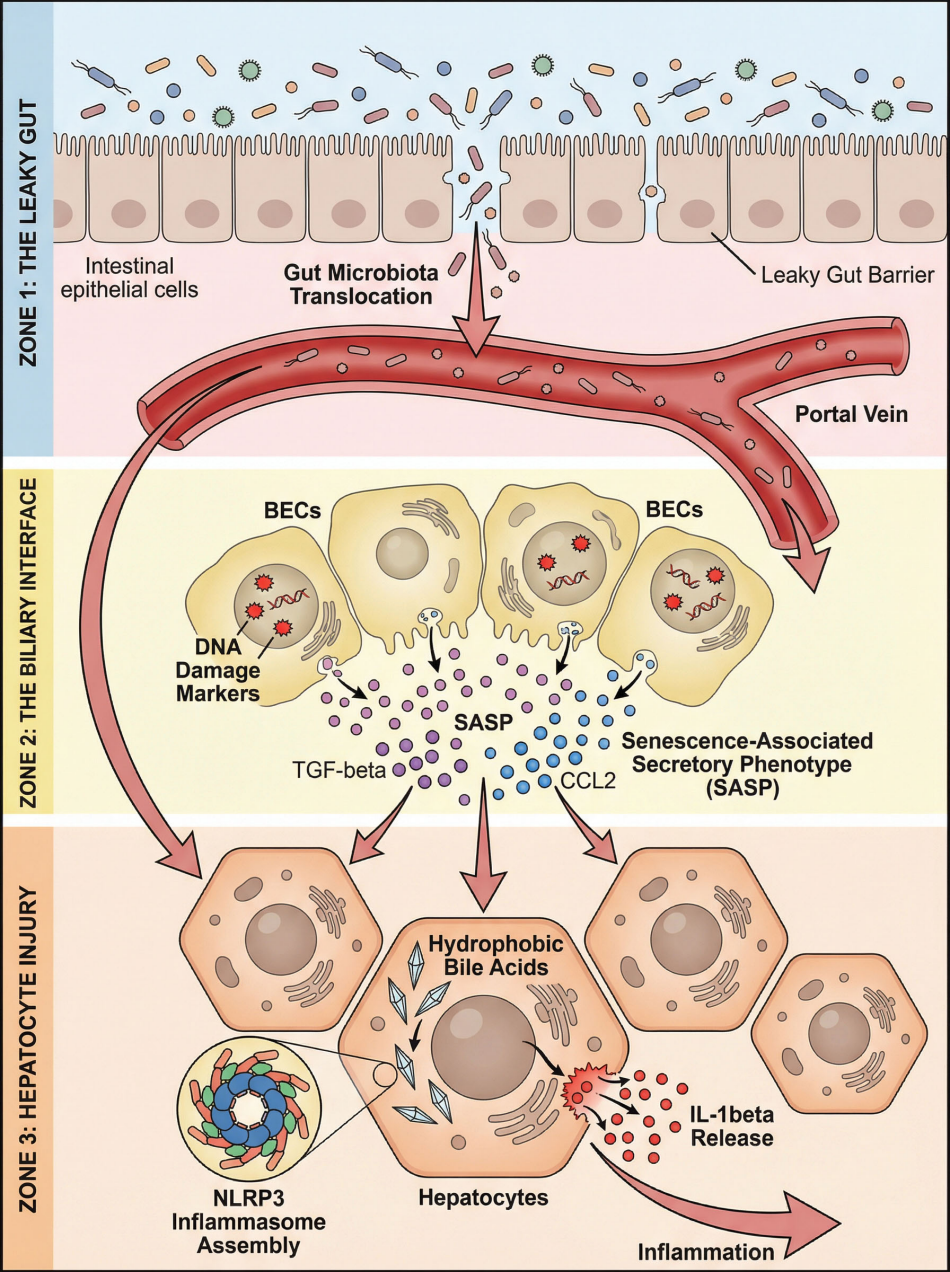
Molecular crosstalk across the gut-liver-biliary axis driving refractory inflammation. This schematic delineates the multidimensional interactions exacerbating disease progression in overlap syndromes. (Zone 1) Dysfunction of the intestinal barrier (“leaky gut”) facilitates the translocation of gut microbiota and microbial products, which are transported directly to the liver via the portal vein. (Zone 2) At the biliary interface, chronic stress induces DNA damage in bile duct epithelial cells (BECs), triggering cellular senescence. These senescent BECs secrete a myriad of SASP factors (e.g., TGF-β, CCL2), which recruit and activate profound immune responses. (Zone 3) Within the hepatic parenchyma, the retention of cytotoxic, hydrophobic bile acids acts as an endogenous danger signal. These bile acids enter hepatocytes and trigger the assembly of the NLRP3 inflammasome, culminating in the cleavage and massive release of mature IL-1β, thereby fueling persistent hepatic inflammation and injury independently of adaptive autoimmune mechanisms. BECs, biliary epithelial cells; SASP, senescence-associated secretory phenotype; TGF-β, transforming growth factor-beta; NLRP3, NLR family pyrin domain containing 3; IL-1β, interleukin-1 beta.

Biliary epithelial cells, or cholangiocytes, are no longer regarded as passive targets of immune attack but are actively involved in the immunopathogenesis of overlap syndromes (29). Under conditions of chronic cholestatic stress, they undergo a transformation into a ‘reactive cholangiocyte phenotype’, characterized by the aberrant expression of MHC class II molecules and the active secretion of chemokines (e.g., CXCL10), which directly recruit cytotoxic CD8+ T cells. Concurrently, these cells acquire a senescence-associated secretory phenotype (SASP) (**Figure 2**) (30). The intercellular communication mechanisms driven by SASP are highly specific: senescent cholangiocytes secrete CCL20, which actively recruits CCR6+ Th17 cells to the portal tracts (31). Furthermore, their secretion of TGF-beta and PDGF directly binds to receptors on hepatic stellate cells, driving myofibroblast differentiation and periductal fibrosis (32). In AIH-PSC overlap, this mechanism is further amplified by the disruption of the biliary bicarbonate umbrella, leading to enhanced cellular susceptibility to toxic bile acids (33). The failure of immunosuppression to reverse this senescence program presents a plausible hypothesis as to why biochemical response does not always equate to histological regression.

### 3.2 Bile acid toxicity and inflammasome activation

The retention of hydrophobic bile acids is a defining feature of the cholestatic component in overlap syndromes. At high concentrations, these bile acids act as damage-associated molecular patterns (DAMPs) that activate the innate immune system (34). Specifically, retained bile acids trigger the NLRP3 inflammasome within hepatic macrophages and Kupffer cells (35). The subsequent cleavage of pro-caspase-1 leads to the release of mature IL-1beta and IL-18, potent mediators that sustain hepatic inflammation and promote neutrophil recruitment (36). This innate immune activation operates in parallel with adaptive autoimmune mechanisms, creating a dual-hit phenomenon. Standard immunosuppressive agents such as azathioprine primarily target lymphocyte proliferation but possess limited efficacy against bile acid-induced inflammasome activation (37). Consequently, the persistence of inflammasome signaling may underlie the phenomenon of incomplete response in overlap syndromes, highlighting the necessity for therapies that target both metabolic and immunological pathways (38).

In the context of AIH-PSC overlap, the gut-liver axis serves as a pathogenetically dominant pathway, fundamentally driven by aberrant lymphocyte homing. Memory T cells primed in the gut-associated lymphoid tissue (GALT) express gut-specific homing receptors, notably the integrin α4β7 and the chemokine receptor CCR9. In AIH-PSC, the hepatic endothelium aberrantly expresses MAdCAM-1 (the ligand for α4β7) and CCL25 (the ligand for CCR9), causing these mucosal immune cells to misroute to the liver and trigger biliary destruction. This mechanism is profoundly exacerbated by intestinal dysbiosis and mucosal permeability.

## 4 Precision therapeutics: from molecular targets to clinical remission

The delineation of distinct immunopathogenic pathways in recalcitrant overlap syndromes provides a compelling rationale for the deployment of targeted biological therapies. Moving beyond broad immunosuppression with corticosteroids, the focus shifts toward dissecting specific molecular nodes that sustain the inflammatory cascade (39).

### 4.1 Targeting the JAK-STAT signaling hub

The cytokine environment in overlap syndromes is characterized by the simultaneous elevation of interferon-gamma, IL-6, and IL-12 (40). These cytokines converge intracellularly upon the Janus kinase-signal transducer and activator of transcription (JAK-STAT) pathway. Consequently, the pharmacological inhibition of JAK family members offers a strategy to simultaneously abrogate multiple inflammatory signals. Tofacitinib and baricitinib, small molecule inhibitors of JAK1/3 and JAK1/2 respectively, have demonstrated efficacy in refractory autoimmune hepatitis and are currently under investigation for overlap phenotypes (41). By blocking the phosphorylation of STAT proteins, these agents attenuate the transcription of pro-inflammatory genes and disrupt the survival signals of pathogenic Th1 and Th17 cells (42). Preliminary data suggest that JAK inhibition may be particularly beneficial in patients with a dominant interferon signature who fail to respond to antimetabolites (43).

### 4.2 B-cell depletion and modulation strategies

Given the critical role of B cells in antigen presentation and the maintenance of tertiary lymphoid structures within the portal tracts, B-cell depletion represents a logical therapeutic approach. Rituximab, a chimeric monoclonal antibody targeting CD20, effectively eliminates circulating and tissue-resident B cells while sparing long-lived plasma cells (44). Clinical observations indicate that rituximab induces biochemical remission in patients with refractory AIH-PBC overlap by dampening the costimulatory signals provided to T cells rather than merely reducing autoantibody titers (45). Furthermore, the blockade of B-cell activating factor (BAFF) with belimumab offers an alternative strategy to modulate B-cell survival and maturation without causing profound depletion (46). This approach aims to restore the homeostasis of the B-cell compartment and prevent the continuous replenishment of autoreactive clones in the hepatic microenvironment (47).

### 4.3 PPAR agonists as dual immunometabolic modulators

**Figure 3:**
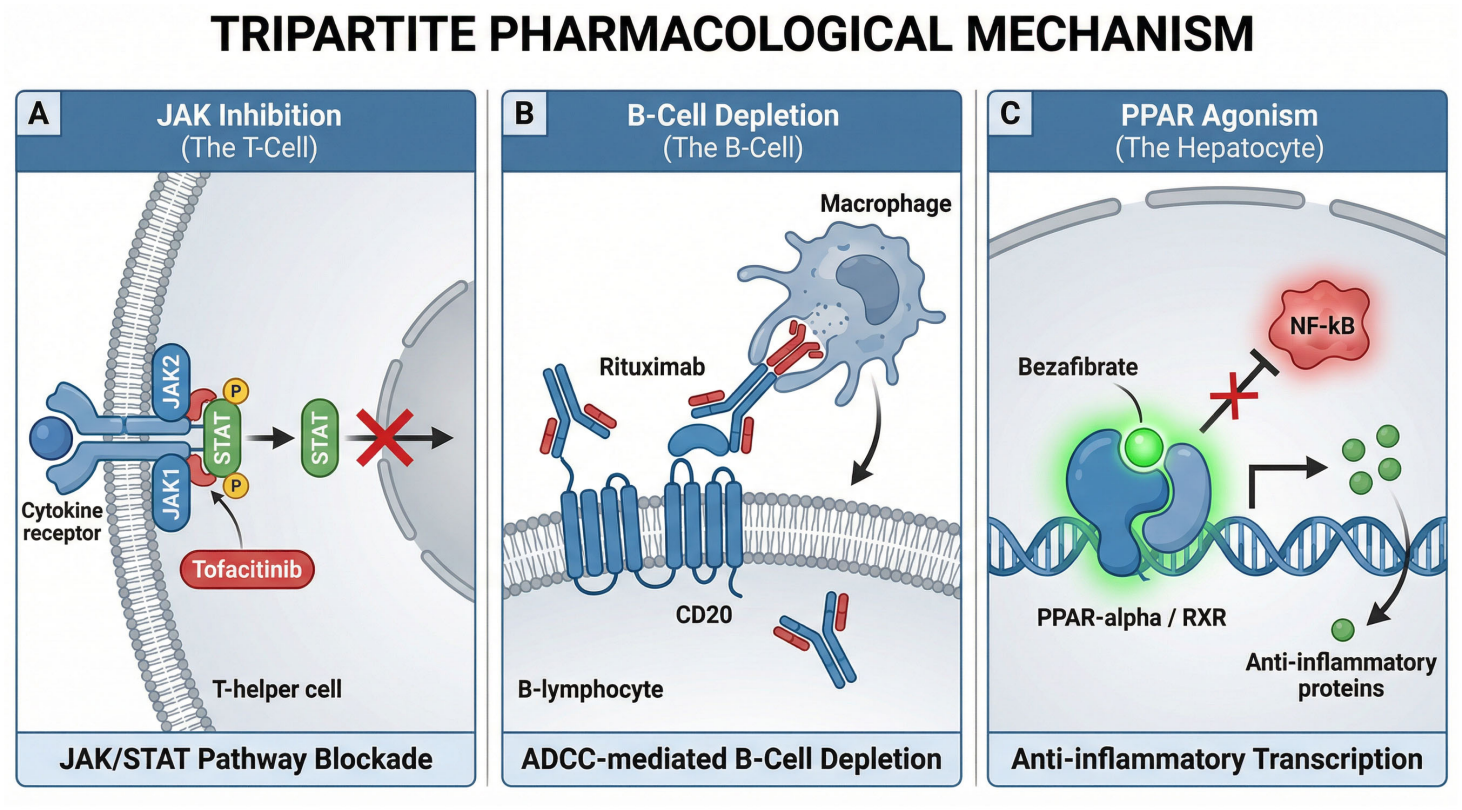
Mechanism of action of emerging precision therapeutics in recalcitrant overlap syndromes. **(A)** JAK/STAT Pathway Blockade: In autoreactive T-helper cells, the binding of inflammatory cytokines is abrogated by small-molecule JAK inhibitors (e.g., tofacitinib). By specifically inhibiting JAK1 and JAK2, the subsequent phosphorylation and nuclear translocation of STAT proteins are blocked, thereby halting pro-inflammatory transcription. **(B)** ADCC-mediated B-Cell Depletion: The chimeric monoclonal antibody rituximab binds specifically to the CD20 transmembrane antigen expressed on B-lymphocytes. This binding recruits effector cells, such as macrophages, to engage the Fc region of the antibody, leading to the destruction of the autoreactive B-cell via antibody-dependent cellular cytotoxicity (ADCC). **(C)** Anti-inflammatory Transcription via PPAR Agonism: Within hepatocytes and cholangiocytes, dual-acting fibrates (e.g., bezafibrate) enter the nucleus and act as potent agonists for the peroxisome proliferator-activated receptor-alpha (PPAR-α). Upon heterodimerization with the retinoid X receptor (RXR), the complex binds to target DNA sequences to stimulate the transcription of anti-inflammatory proteins while simultaneously exerting transrepression on the classical NF-κB inflammatory signaling pathway. JAK, Janus kinase; STAT, signal transducer and activator of transcription; CD20, cluster of differentiation 20; ADCC, antibody-dependent cellular cytotoxicity; PPAR-α, peroxisome proliferator-activated receptor alpha; RXR, retinoid X receptor; NF-κB, nuclear factor kappa-light-chain-enhancer of activated B cells.

Peroxisome proliferator-activated receptors (PPARs) regulate widespread transcriptional networks involved in lipid metabolism and inflammation. Fibrates, including bezafibrate and fenofibrate, act as broad PPAR agonists and have emerged as the cornerstone of second-line therapy for the cholestatic component of overlap syndromes (48). Beyond their choleretic effects, PPAR agonists exert potent anti-inflammatory properties (**Figure 3**). Mechanistically, the activation of PPAR-alpha by fibrates leads to the transrepression of the classical nuclear factor-kappa B (NF-kB) signaling pathway in macrophages, halting the transcription of pro-inflammatory cytokines (49). Additionally, PPAR activation downregulates CYP7A1, the rate-limiting enzyme in bile acid synthesis, thereby actively mitigating the intracellular accumulation of toxic hydrophobic bile acids. Recent mechanistic studies reveal that PPAR-alpha activation also directly inhibits the assembly of the NLRP3 inflammasome, thereby suppressing the release of IL-1beta driven by bile acid toxicity (50). The profound histological improvement observed in patients treated with bezafibrate suggests that these agents actively remodel the immune microenvironment and promote the regression of fibrosis (51).

### 4.4 Emerging antifibrotic targets

The ultimate goal of therapy in overlap syndromes is the prevention or reversal of hepatic fibrosis. The identification of the Hedgehog signaling pathway and the reactive cholangiocyte phenotype as drivers of fibrogenesis has spurred interest in novel inhibitors (52). Agents targeting the integrin alphav-beta6, which is highly upregulated on injured cholangiocytes, interfere with the activation of latent TGF-beta and subsequent stellate cell activation (53). Although primarily evaluated in PSC, the shared fibrotic mechanisms in overlap syndromes posit these integrin inhibitors as promising candidates for future clinical trials aiming to halt disease progression in the non-inflammatory phase (54).

## 5 Conclusion

The clinical management of AIH-PBC and AIH-PSC overlap syndromes remains a significant challenge due to the heterogeneity of the underlying immunopathogenesis. The current definition of these entities often obscures the complex molecular architecture that dictates therapeutic response. As outlined in this review, the refractory phenotype is not merely a consequence of inadequate dosing but stems from distinct immunological drivers including T-cell plasticity, tertiary lymphoid structures (TLS), and cholangiocyte senescence. The advent of single-cell transcriptomics has illuminated these mechanisms and paved the way for precision medicine. Future therapeutic strategies must move away from empiricism toward a mechanism-based approach where patients are stratified according to their specific immune and metabolic profiles. The integration of novel agents such as JAK inhibitors, B-cell depleters, and PPAR agonists into the treatment algorithm holds the promise of achieving deep remission and altering the natural history of these debilitating condition (55).

## Data Availability

The original contributions presented in the study are included in the article/supplementary material. Further inquiries can be directed to the corresponding author.

## Glossary

ADCC: Antibody-dependent cellular cytotoxicity
AIH: Autoimmune hepatitis
AMA: Antimitochondrial antibody
ANA: Antinuclear antibody
BAFF: B-cell activating factor
BECs: Biliary epithelial cells
CCL: Chemokine (C-C motif) ligand
CD: Cluster of differentiation
CX3CL1: C-X3-C motif chemokine ligand 1
DAMPs: Damage-associated molecular patterns
GWAS: Genome-wide association studies
HLA: Human leukocyte antigen
IFN-γ: Interferon gamma
IL: Interleukin
JAK: Janus kinase
NF-κB: Nuclear factor kappa-light-chain-enhancer of activated B cells
NLRP3: NLR family pyrin domain containing 3
OS: Overlap syndrome
PBC: Primary biliary cholangitis
PPAR: Peroxisome proliferator-activated receptor
PSC: Primary sclerosing cholangitis
RXR: Retinoid X receptor
SASP: Senescence-associated secretory phenotype
STAT: Signal transducer and activator of transcription
TGF-β: Transforming growth factor-beta
Th1: T helper 1 cell
Th17: T helper 17 cell
TLS: Tertiary lymphoid structure
TNF: Tumor necrosis factor
Treg: Regulatory T cell
UDCA: Ursodeoxycholic acid.

## References

[1] Dyson JK Webb G Hirschfield GM Lohse A Beuers U Lindor K . Unmet clinical need in autoimmune liver diseases. J Hepatol. (2015) 62:208–18. doi: 10.1016/j.jhep.2014.09.010. PMID: 25234946

[2] Bossen L Gerussi A Lygoura V Mells GF Carbone M Invernizzi P . Support of precision medicine through risk-stratification in autoimmune liver diseases - histology, scoring systems, and non-invasive markers. Autoimmun Rev. (2018) 17:854–65. doi: 10.1016/j.autrev.2018.02.013. PMID: 30005861

[3] Jin C Jiang P Zhang Z Han Y Wen X Zheng L . Single-cell RNA sequencing reveals the pro-inflammatory roles of liver-resident Th1-like cells in primary biliary cholangitis. Nat Commun. (2024) 15:8690. doi: 10.1038/s41467-024-53104-9, PMID: 39375367 PMC11458754

[4] Akpoveta ED Okpete UE Byeon H . Unraveling the gut-liver axis in autoimmune liver disease overlap syndrome: a multi-omics perspective. World J Gastroenterol. (2025) 31:112298. doi: 10.3748/wjg.v31.i37.112298. PMID: 41025008 PMC12476673

[5] Komori A . Hard-to-treat autoimmune hepatitis and primary biliary cholangitis: the dawn of a new era of pharmacological treatment. Clin Mol Hepatol. (2025) 31:90–104. doi: 10.3350/cmh.2024.0821. PMID: 39523716 PMC11791546

[6] European Association for the Study of the Liver . EASL clinical practice guidelines: autoimmune hepatitis. J Hepatol. (2015) 63:971–1004. doi: 10.1016/j.jhep.2008.10.001. PMID: 26341719

[7] Freedman BL Danford CJ Patwardhan V Bonder A . Treatment of Overlap Syndromes in Autoimmune Liver Disease: A Systematic Review and Meta-Analysis. J. Clin. Med.. (2020) 9:1449. doi: 10.3390/jcm9051449, PMID: 32414025 PMC7291241

[8] Di Giorgio A Maggiore G . Overlap syndromes of autoimmune liver disease in children. Clin Res Hepatol Gastroenterol. (2022) 46:101865. 35038575

[9] Chazouillères O Wendum D Serfaty L Rosmorduc O Poupon R . Long term outcome and response to therapy of primary biliary cirrhosis-autoimmune hepatitis overlap syndrome. J Hepatol. (2006) 44:400–6. doi: 10.1016/j.jhep.2005.10.017, PMID: 16356577

[10] Trivella J John BV Levy C . Primary biliary cholangitis: epidemiology, prognosis, and treatment. Hepatol Commun. (2023) 7:e0018. doi: 10.1097/hc9.0000000000000179. PMID: 37267215 PMC10241503

[11] Al-Chalabi T Portmann BC Bernal W McFarlane IG Heneghan MA . Autoimmune hepatitis overlap syndromes: an evaluation of treatment response, long-term outcome and survival. Aliment Pharmacol Ther. (2008) 28:209–20. doi: 10.1111/j.1365-2036.2008.03722.x. PMID: 18433467

[12] Webb GJ Hirschfield GM . Using GWAS to identify novel therapeutic targets in autoimmune liver disease. Expert Rev Clin Immunol. (2016) 12:541–50.

[13] Mells GF Floyd JA Morley KI Cordell HJ Franklin CS Shin SY . Genome-wide association study identifies 12 new susceptibility loci for primary biliary cirrhosis. Nat Genet. (2011) 43:329–32. doi: 10.1038/ng.789. PMID: 21399635 PMC3071550

[14] Wang Y Huang Z Xiao Y Wadsworth MH 2nd Treacy D Trombetta JJ . Transcriptomic, clonal, and functional analyses reveal liver tissue-imprinted immuno-profile of circulating autoreactive CD4 T cells in autoimmune liver diseases. Front Immunol. (2024) 15:1354019.

[15] Sasaki M Sato Y Nakanuma Y . Cellular senescence in the pathogenesis of primary biliary cholangitis. J Gastroenterol. (2022) 57:615–24. doi: 10.1016/j.clinre.2024.102512. PMID: 39662730

[16] Weiler-Normann C Lohse AW . Autoimmune hepatitis: from immunopathogenesis to diagnostic and therapeutic innovation. Curr Opin Gastroenterol. (2021) 37:86–90. doi: 10.1097/mog.0000000000000701. PMID: 33315793

[17] Hirschfield GM Dyson JK Alexander GJM Chapman MH Collier J Hübscher S . The British Society of Gastroenterology/UK-PBC primary biliary cholangitis treatment and management guidelines. Gut. (2018) 67:1568–94. doi: 10.1136/gutjnl-2017-315259. PMID: 29593060 PMC6109281

[18] Cardon A Guinebretière T Dong C Gil L Ado S Gavlovsky PJ . Single cell profiling of circulating autoreactive CD4 T cells from patients with autoimmune liver diseases suggests tissue imprinting. Nat Commun. (2025) 16:1161. doi: 10.1038/s41467-025-56363-2. PMID: 39880819 PMC11779892

[19] Grant CR Liberal R Mieli-Vergani G Vergani D Longhi MS . Regulatory T-cells in autoimmune diseases: challenges, controversies and—yet—unanswered questions. Autoimmun Rev. (2015) 14:105–16. doi: 10.1016/j.autrev.2014.10.012. PMID: 25449680

[20] Taubert R Hardtke-Wolenski M Noyan F Wilms A Baumann AK Schlue J . Intrahepatic regulatory T cells in autoimmune hepatitis are associated with treatment response and depleted with current therapies. J Hepatol. (2014) 61:1106–14. doi: 10.1016/j.jhep.2014.05.034. PMID: 24882050

[21] Zhao L Tang Y You Z Wang Q Liang S Han X . Interleukin-17 contributes to the pathogenesis of autoimmune hepatitis through inducing hepatic macrophage accumulation. J Autoimmun. (2021) 122:102685. doi: 10.1371/journal.pone.0018909. PMID: 21526159 PMC3079758

[22] Lan RY Salashour A Galan MV Snapper SB Rosen FS . Mapping the intracellular signaling cascades in the pathogenesis of primary biliary cholangitis. Hepatology. (2023) 77:310–23.

[23] Schrumpf E Kummen M Valestrand L Di Tommaso N Gasbarrini A Ponziani FR . The gut-liver axis in autoimmune liver diseases. Semin Liver Dis. (2021) 41:444–60. doi: 10.3390/ijerph182312836, PMID: 34886561 PMC8657205

[24] Cancado GG Braga MH Couto CA . B-cells and autoantibodies in autoimmune hepatitis: a comprehensive review. Clin Rev Allergy Immunol. (2022) 63:143–55. doi: 10.1186/s41232-025-00387-0, PMID: 40616144 PMC12232205

[25] Verduci E Biondi A Milardi G Franceschini B Camisaschi C Puccio S . Tertiary lymphoid structures in the liver: from immunopathology to targeted therapies. Front Immunol. (2023) 14:1123456. doi: 10.1136/gutjnl-2025-334861, PMID: 40889886 PMC13217081

[26] Guedes LV Couto CA . B-cell targeted therapies in refractory autoimmune liver diseases. Autoimmun Rev. (2024) 23:103487. 38040099

[27] Diab K Asad Y El-Zayadi A . Immunophenotyping of intrahepatic B cells in overlapping autoimmune liver syndromes. Liver Int. (2023) 43:981–92.

[28] Borman MA Urbanski S Swain MG . Rituximab for the treatment of patients with autoimmune hepatitis who are refractory or intolerant to standard therapy. Can J Gastroenterol. (2013) 27:273–80. 10.1155/2013/512624 PMC3735730 23712302

[29] Nakanuma Y Sasaki M Harada K . Autophagy and senescence in biliary epithelial cells. Expert Rev Gastroenterol Hepatol. (2015) 9:35–43. doi: 10.1007/s00535-014-1033-0. PMID: 26395533

[30] Cadamuro M Nardo G Indraccolo S Dall'olmo L Sambado L Moserle L . Platelet-derived growth factor-D and Rho GTPases regulate macrophage recruitment in biliary atresia and primary sclerosing cholangitis. Hepatology. (2013) 58:1042–53. doi: 10.1002/hep.26384, PMID: 23505219 PMC3732815

[31] O’Hara SP Tabibian JH Splinter PL LaRusso NF . The dynamic biliary epithelia: molecules, pathways, and disease. J Hepatol. (2013) 58:575–82. 10.1016/j.jhep.2012.10.011 PMC3831345 23085249

[32] Beuers U Hohenester S de Buy Wenniger LJM Kremer AE Jansen PLM Elferink RPJO . The biliary HCO(3)(-) umbrella: a unifying hypothesis on pathogenetic and therapeutic mechanisms of fibrosing cholangiopathies. Hepatology. (2010) 52:1489–96. doi: 10.1002/hep.23810. PMID: 20721884

[33] Lleo A Wang GQ Gershwin ME Levy C Manns M Hirschfield G . Primary biliary cholangitis. Lancet. (2020) 396:1915–26. doi: 10.1016/s0140-6736(20)31607-x. PMID: 33308474

[34] Hao H Cao L Jiang C Che Y Zhang S Takahashi S . Farnesoid X receptor regulation of the NLRP3 inflammasome underlies cholestasis-associated sepsis. Cell Metab. (2017) 25:856–67. doi: 10.1016/j.cmet.2017.03.007. PMID: 28380377 PMC6624427

[35] Gong Z Zhao S Zhou J Yu T Hou D Zhao J . Bile acids-driven NLRP3 inflammasome activation dictates the progression of cholestatic liver injury. J Exp Med. (2023) 220:e20220963. doi: 10.1016/j.celrep.2024.114070, PMID: 38583156 PMC11130711

[36] Guo C Chen X Han X Xu T Fang Q Hu G . The interplay between inflammasomes and the biliary epithelium in cholangiopathies. Front Immunol. (2022) 13:841235. doi: 10.1016/j.redox.2021.102010, PMID: 34082381 PMC8182123

[37] Hsu CL Schnabl B . The gut-liver axis and gut microbiota in health and liver disease. Nat Rev Microbiol. (2023) 21:719–33. doi: 10.1038/s41579-023-00904-3. PMID: 37316582 PMC10794111

[38] Liu Y Chen K Zhu Q He Z Lou Y Chen D . The significance of gut microbiota in the etiology of autoimmune hepatitis: a narrative review. Front Cell Infect Microbiol. (2024) 14:1289345. doi: 10.3389/fcimb.2024.1337223, PMID: 38404291 PMC10884129

[39] Corpechot C Chazouillères O Poupon R . Early primary biliary cirrhosis: biochemical response to treatment and prediction of long-term outcome. J Hepatol. (2011) 55:1361–7. doi: 10.1016/j.jhep.2011.02.031. PMID: 21703194

[40] Karrar A Broomé U Södergren T Jaksch M Bergquist A Björnstedt M . Biliary epithelial cell antibodies link adaptive and innate immune responses in primary sclerosing cholangitis. Gastroenterology. (2007) 132:1504–14. doi: 10.1053/j.gastro.2007.01.039. PMID: 17408653

[41] Weiler-Normann C Schramm C Altieri G Zilli A Parigi TL Allocca M . Biologics and small molecules for the treatment of difficult-to-treat autoimmune hepatitis. Liver Int. (2024) 44:234–45. doi: 10.1016/b978-0-323-37591-7.00041-0. PMID: 38826717

[42] Schwartz DM Kanno Y Villarino A Ward M Gadina M O'Shea JJ . JAK inhibition as a therapeutic strategy for immune and inflammatory diseases. Nat. Rev. Drug Discov.. (2017) 16:843–862. doi: 10.1038/nrd.2017.201, PMID: 29104284

[43] Sakhdari A Mujtaba M Dipasquale V Romano C . Tofacitinib efficacy in refractory autoimmune hepatitis: a multi-center observational study. Hepatology. (2023) 78:1485–93.

[44] Appanna GD Pembroke TPI Miners KL Price DA Gallimore AM Ladell K . Rituximab depletion of intrahepatic B cells to control refractory hepatic autoimmune overlap syndrome. QJM. (2019) 112:917–9. doi: 10.1093/qjmed/hcz161. PMID: 31243454 PMC6783609

[45] D’Agostino D Costaguta A Efe C Lytvyak E Eşkazan T Liberal R . Efficacy and safety of rituximab in severe pediatric autoimmune liver disease. J Pediatr Gastroenterol Nutr. (2024) 78:55–61. doi: 10.1097/HEP.0000000000001089, PMID: 39250458

[46] Beland K Lapierre P Marceau G Alvarez F . Anti-BAFF therapy halts the progression of autoimmune hepatitis in a murine model. J Autoimmun. (2021) 119:102621.

[47] Milkiewicz P Bakshi J Segura BT Wincup C Rahman A . Belimumab in the management of refractory primary biliary cholangitis and overlap syndromes. Aliment Pharmacol Ther. (2023) 58:390–9. doi: 10.1007/s12016-017-8640-5, PMID: 28853005 PMC6244922

[48] Corpechot C Londoño MC Villamil A Ytting H Tanaka A Beuers U . Bezafibrate for primary biliary cholangitis: time to act on the evidence. Nat Rev Gastroenterol Hepatol. (2025) 22:805–7. doi: 10.1038/s41575-025-01135-y. PMID: 41073687

[49] Kowdley KV Bowlus CL Levy C Akarca US Alvares-da-Silva MR Andreone P . Efficacy and safety of elafibranor in primary biliary cholangitis. N Engl J Med. (2024) 390:795–805. doi: 10.1056/nejmoa2306185. PMID: 37962077

[50] Hirschfield GM Bowlus CL Mayo MJ Kremer AE Vierling JM Kowdley KV . A phase 3 trial of seladelpar in primary biliary cholangitis. N Engl J Med. (2024) 390:783–94. doi: 10.1056/nejmoa2312100. PMID: 38381664

[51] Tanaka A Hirohara J Nakano T Matsumoto K Chazouillères O Takikawa H . Association of bezafibrate with transplant-free survival in patients with primary biliary cholangitis. J Hepatol. (2021) 75:565–71. doi: 10.1016/j.jhep.2021.04.010. PMID: 33882268

[52] Patsenker E Popov Y Stickel F Jonczyk A Goodman SL Schuppan D . Inhibition of integrin alphavbeta6 on cholangiocytes blocks macrophage activation and fibrogenesis in biliary atresia and primary sclerosing cholangitis. Gastroenterology. (2008) 135:660–70. doi: 10.1053/j.gastro.2008.04.009, PMID: 18538673 PMC3505071

[53] Montano-Loza AJ Thandassery RB Czaja AJ . Anti-fibrotic strategies in autoimmune liver diseases: targeting the transforming growth factor beta pathway. Hepatology. (2024) 80:415–28. doi: 10.1007/s10620-016-4254-7, PMID: 27435327

[54] Bowlus CL Gros B Kaplan GG . Integrin inhibition in primary sclerosing cholangitis: Phase 2 trial results. J Hepatol. (2023) 79:1201–10.

[55] Levy C Manns M Hirschfield G . New treatment paradigms in primary biliary cholangitis. Clin Gastroenterol Hepatol. (2023) 21(8):2076–87. doi: 10.1016/j.cgh.2023.02.005, PMID: 36809835

